# Brachydactyly type B: a rare case report and literature review

**DOI:** 10.1093/jscr/rjae376

**Published:** 2024-05-31

**Authors:** Mohannad Natheef AbuHaweeleh, Mohamed Badie Ahmed, Fatima Saoud Al-Mohannadi, Massoud Daw Mohamed, Abeer AlSherawi

**Affiliations:** College of Medicine, QU Health, Qatar University, P.O. Box 2713, Doha, Qatar; College of Medicine, QU Health, Qatar University, P.O. Box 2713, Doha, Qatar; Plastic Surgery Department, Hamad General Hospital, Hamad Medical Corporation, P.O. Box 3050, Doha, Qatar; Plastic Surgery Department, Hamad General Hospital, Hamad Medical Corporation, P.O. Box 3050, Doha, Qatar; Plastic Surgery Department, Hamad General Hospital, Hamad Medical Corporation, P.O. Box 3050, Doha, Qatar; Plastic Surgery Department, Hamad General Hospital, Hamad Medical Corporation, P.O. Box 3050, Doha, Qatar

**Keywords:** brachydactyly type B, hypoplastic digits, brachymetatarsia, congenital anomaly

## Abstract

Brachydactyly is a genetic condition leading to shortened or absent digits in hands or feet. It can occur independently or as part of syndromes. This case focuses on Brachydactyly type B, the rarest form. An 8-month-old from the Philippines was referred due to a missing third toe. Examination revealed a hypoplastic left third toe. X-rays confirmed the finding. Treatment options were discussed, including conservative therapy and follow up. Diagnosis involved history, examination, and imaging. Prenatal diagnosis is limited for isolated cases but useful for syndromic forms if a family mutation is known. Prognosis varies depending on the severity and associated syndromes. Currently there is no definitive treatment; management involves genetic counseling and therapy. Due to limited cases, Type B is underreported, highlighting the need for more research into its genetics.

## Introduction

Brachydactyly is a rare congenital malformation characterized by disproportionally short digits [1, 2]. This manifestation can present as an isolate defect or part of an underlying syndrome [1, 2]. Brachydactyly can coexist with other digit malformations including syndactyly, polydactyl, reduction defect, or symphalangism [2]. Depending on affected bone, there are eight main types of isolated brachydactyly including A, B, C, D, E, Brachy metatarsus IV, Sugarman brachydactyly, and Kirner deformity [2]. Type B is considered one of the rare subtypes of brachydactyly [1]. Brachydactyly Type B1 is considered the most severe type with an autosomal dominant pattern of inheritance [1, 2], high penetrance, and variable expressivity. Brachydactyly Type B1 is associated with receptor kinase-like orphan receptor 2 (ROR2) gene mutation on chromosome 9q22.31 [1, 2]. The specific phenotypical manifestation of Brachydactyly Type B1 is usually hypoplastic or aplastic distal phalanges and nails of digits 2 through 5 in the hand or feet [2, 3]. Currently, there is no necessity for treatment if physiological functions of digits are intact, otherwise cosmetic surgery can be indicated to fix the phenotypical appearance [4, 5]. We aim to present this rare case as there is scarce literature available on Brachydactyly Subtype B, primarily due to its rarity. Furthermore, there are no reported images depicting brachydactyly with the absence of the middle and distal phalanges of the left third toe, making this case particularly noteworthy. To our knowledge, this is the first reported case of a rare clinical manifestation of Brachydactyly type B in a male patient of Asian ancestry.

## Case report

An 8-month-old male from Philippines referred from primary healthcare center presented to the plastic surgery clinic with his mother requesting for second opinion on a missing third toe on the left foot since birth. He is the fourth child with no history of similar conditions in the family.

Growth chart for weight shows 8 kg at 15th percentile, while growth chart for length shows 65.5 cm at 3rd percentile. Patient is on cholecalciferol (Vitamin D3) 400 international units PO daily. According to records, patient is up to date with vaccination schedule. No qualifying family history data was available. No known allergies. Past medical history was positive for fever for one day and skin rash for 2 days at 6 months of age with no cough, no runny nose, no sore throat, no difficulty breathing, no vomiting, and no diarrhea. Past medical history suggestive of hand foot and mouth disease. No past surgical history.

**Figure 1:**
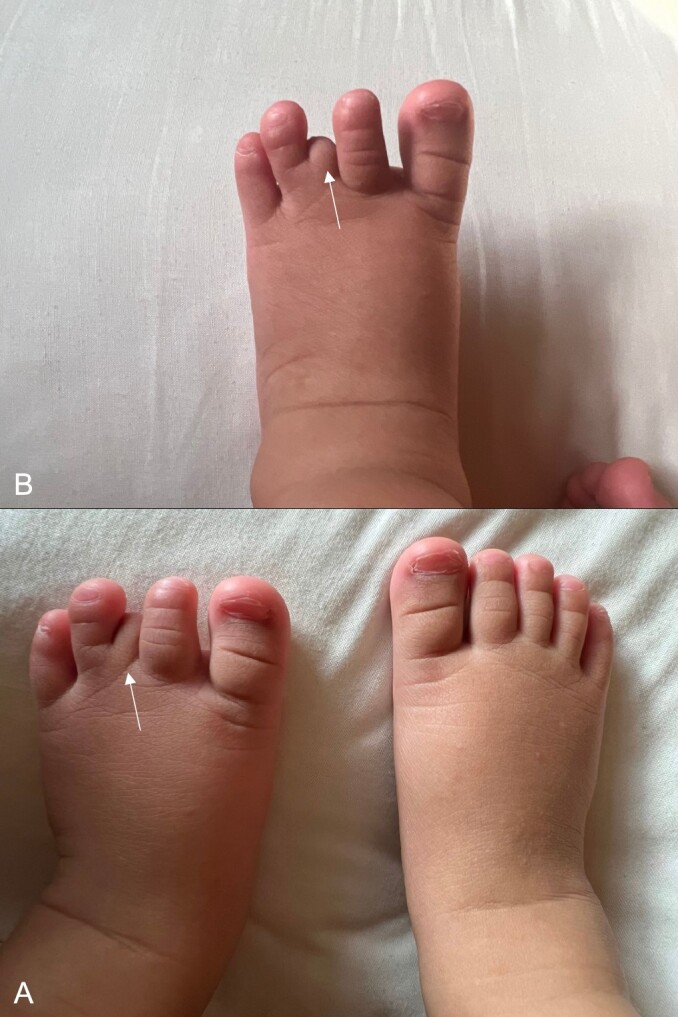
(A) Bilateral feet with normal right foot and left foot (arrow). (B) Hypoplastic left third toe with no visualization of the whole digit and absence of toenail (arrow).

Physical exam showed brachymetatarsia—hypoplastic left third toe with no visualization of the whole digit and absence of toenail (Fig. 1) The rest of the hand and right foot digits appear unremarkable. Hypoplastic left third toe appears nontender, no redness, no swelling, no discharge, and no cysts.

**Figure 2:**
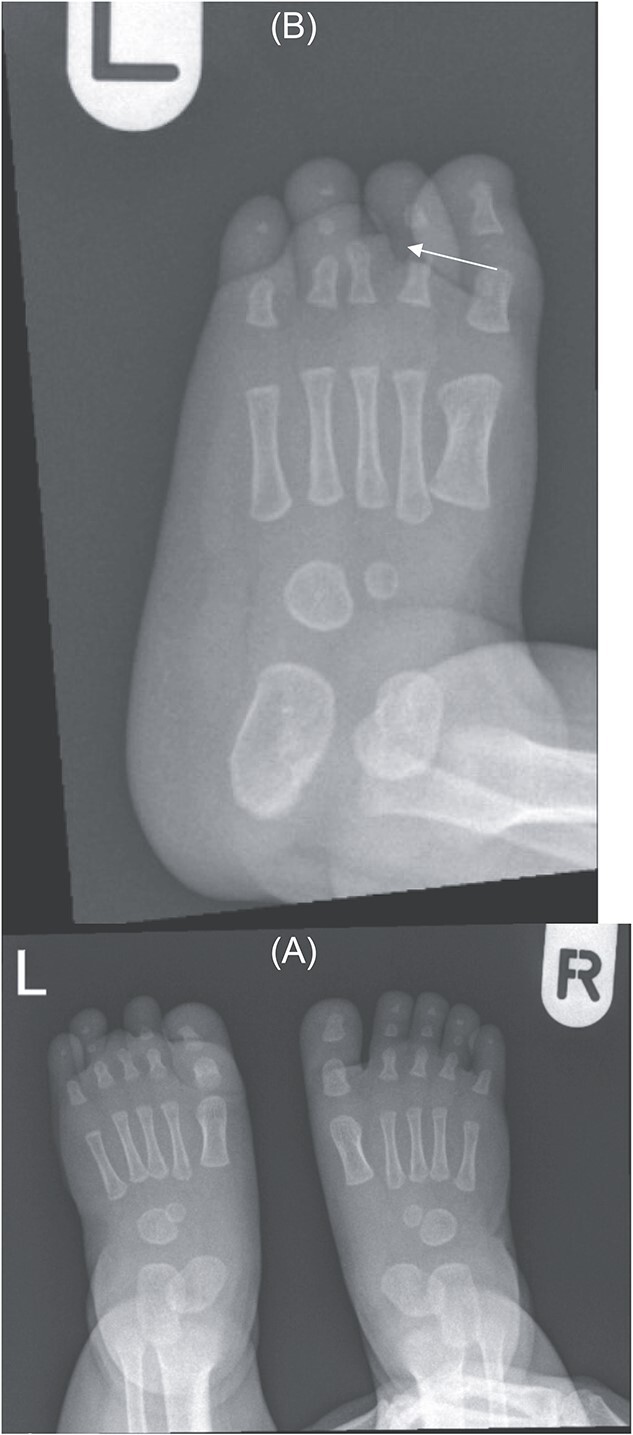
(A) Bilateral feet X-ray with normal right foot bone anatomy and left foot X ray. (B) No visualization of third toe middle and distal phalanges (arrow).

Foot X-ray confirms hypoplastic left third toe with no visualization of middle and distal phalanges — suggesting a very rare variant of brachydactyly (type B) (Fig. 2).

Conservative therapy and follow up after one year were discussed and agreed upon with patient’s mother. Elective cosmetic surgery may be offered later if indicated.

## Discussion

Brachydactyly is an inherited genetic anomaly characterized by the shortening or absence of digits in the hand or foot. This rare condition can have isolated forms, or it can be a feature of a syndromic disease. Brachydactyly can have many types, summarized in Table 1, the rarest form is Brachydactyly type B which was described in this case report. Brachydactyly Type B is underreported in the literature due to the rarity of the cases, thereby there is a need to further contribute to related research to this condition to further understand its genetic components. Brachydactyly Type B is autosomal dominant if presented in an isolated fashion. However, it can present as part of an autosomal recessive condition such as severe Robinow Syndrome which can have multiple additional dysmorphic features such as mesolimbic short stature, abnormalities in the face, ribs, vertebra, as well as malformations of the limb, thorax, and genitalia [6]. Milder form of Robinow syndrome is autosomal dominant which can present with umbilical hernia and supernumerary teeth [6]. Brachydactyly Type B can be further divided into types B1 and B2 [5]. Brachydactyly Type B1 is due to a mutation in the *ROR2* gene. *ROR2* gene translates into a receptor kinase-like protein that is involved in cellular growth and division. It is associated with other cellular signaling proteins such as Wingless-related Integration Site (Wnt). Brachydactyly Type B2 is due to a *NOG* gene mutation [5]. *NOG* gene provides information to form noggin protein which regulates bone morphogenetic proteins by covalently binding to them and inhibiting their receptor binding [7].

**Table 1:** Various types of brachydactyly with their respective digits and genes involved.

**Type**	**Digits affected**	**Gene**
A1 (Farabee type Brachydactyly)	Middle phalanges of all digits, rending them to be undeveloped or fused with distal phalanges	Mutation of Indian hedgehog gene (*IHH*)
A2 (Mohr-Wreidt type Brachydactyly)	Middle phalanges of the index finger and/or 2nd toe	Mutations in different genes including: bone protein morphogenetic protein receptor type B1 (*BMPR1B*), bone morphogenetic protein 2 (*BMP2*), growth/differentiation factor 5 (*GDF5*)
A3 (Brachymesophalangy V or Brachydactyly-clinodactyly)	Middle phalanx of the fifth (little) finger	Mutation of Homeobox protein D13 (*HOXD13*) gene
A4 (Brachymesophalangy II and V, Temtamy Type Brachydactyly)	Middle phalanges of the 2nd and 5th digits	Mutation of *HOXD13* gene
A5 (Can be considered under Brachydactyly Type B)	Middle phalanges of digits 2 to 5, associated with nail dysplasia	
B1	Distal phalanges digits 2 to 5, associated with aplasia of fingernails, the middle phalanges can also be hypoplastic	Mutation of *ROR2* gene
B2		Mutation of *NOG* gene
C (Brachydactyly with Hyperphalangism or Haws Type)	Brachymesophalangy of digits 2, 3, and 5 with hyperphalangy of the 2nd and 3rd digits and shortening of the 1st metacarpal	Mutation of *GDF5* and *HOXD13* gene
D (Stumb Thumb)	Distal phalanx of the 1st digit	Mutation of *HOXD13* gene
E	Metacarpals and metatarsals	Mutation of *HOXD13* gene
Brachy metatarsus IV	Fourth metatarsi, bilaterally or unilaterally	
Sugarman brachydactyly	Hallux, causing it to set dorsal and proximal to its usual position	
Kirner deformity (Dystelephalngy)	Bilateral fifth fingers (little), causing it to have radial bowing of distal phalanx	

Isolated Brachydactyly has other various forms that usually affect different parts of the digits. Most isolated brachydactyly are inherited in an autosomal dominant fashion. Brachydactyly type A1, also known as Farabee type brachydactyly, is due to a mutation in the Indian hedgehog gene (*IHH*) [2], it mainly affects the middle phalanges of all digits rending them to be undeveloped or fused with distal phalanges. Brachydactyly type A2, also known as Mohr-Wreidt type brachydactyly, is due to mutations in different genes including bone protein morphogenetic protein receptor type B1 (*BMPR1B*) [2], bone morphogenetic protein 2 (*BMP2*), or growth/differentiation factor 5 (*GDF5*) [2]. Brachydactyly type A2 mainly affects the middle phalange of the index finger and/or 2^nd^ toe [2]. Recent evidence showed that Homeobox protein D13 (*HOXD13*) mutation is associated with Brachydactyly types A3, A4, D, and E [8]. Brachydactyly type A3, also called Brachymesophalangy V or Brachydactyly-clinodactyly, usually affects middle phalanx of the fifth (little) finger [2]. Brachydactyly type A4, also called Brachymesophalangy II and V, Temtamy Type Brachydactyly, usually affects the middle phalanx of the 2^nd^ and 5^th^ digits. Brachydactyly Type A5 usually affects middle phalanges of digits 2 to 5 and is associated with nail dysplasia [2]. It is thought that this subtype can be considered under Brachydactyly Type B [2]. Brachydactyly Type B usually affects distal phalanges digits 2 to 5 associated with aplasia of fingernails, the middle phalanges can also be hypoplastic [2]. Brachydactyly type C, also called Brachydactyly with Hyperphalangism or Haws Type, is due to a mutation in *GDF5* [2, 9]. It usually manifests as brachymesophalangy of digits 2, 3, and 5 with hyperphalangy of the second and third digits and shortening of the first metacarpal [2]. Brachydactyly Type D, also known as Stumb Thumb, usually affects the distal phalanx of the first digit [2]. Brachydactyly Type E usually affects the metacarpals and metatarsals; however it may have overlapping features with Brachydactyly Type D [2]. Brachymetatarsus IV usually affects the fourth metatarsi bilaterally or unilaterally. As distinction from Brachydactyly Type E, Brachymetatarsus IV does not affect the metacarpals [2]. Sugarman brachydactyly mainly affects the hallux causing it to set dorsal and proximal to its usual position [2]. Kirner deformity, also called dystelephalngy, usually affects bilateral fifth fingers (little) causing it to have radial bowing of distal phalanx [2].

Diagnosing brachydactyly depends on the proper history, physical exam, and radiological imaging [1]. Prenatal diagnosis may not be that helpful in early diagnosis of isolated congenital brachydactyly, while in the syndromic forms, it may be of use [2]. Abnormal phalanges may not be visualized using fetal ultrasound at early stages, however, at Weeks 11 and 14 of gestation, chorionic villous sampling and amniocentesis, respectively, may provide antenatal diagnosis if mutation in the family history is known [2]. Prognosis of brachydactyly is solely dependent on the type of malformation and its influence on the hand’s function. If brachydactyly appears with an underlying syndrome, the prognosis depends on the other associated features [2]. To date, there is no definite treatment for brachydactyly as it does not deem necessary or affect the quality of life. Genetic counseling and testing might be of aid to indicate the pattern of inheritance and determine the affected gene. Physical and/or occupation therapy may be implied to improve digits’ physiological function [1, 2]. Plastic surgery can be indicated for cosmetic purposes. Limb lengthening surgery, such as Ilizarov technique, can be of potential application for certain variants of brachydactyly [10]. There are no reported studies on applying gene therapy as a method of intervention for brachydactyly. To our knowledge, this is the first case report to report Isolated Brachydactyly Type B in an individual with Asian ancestry and at this early age of diagnosis. Other case reports of brachydactylies involved individuals from Japan [11], India [12], and China [3, 9, 13].

## Data Availability

All data generated or analyzed during this study are included in this article. Further enquiries can be directed to the corresponding author.
